# Supplementary figures and images for: Divergent functions of two clades of flavodoxin in diatoms mitigate oxidative stress and iron limitation
Source: eLife. 2023 Jun 6;12:e84392. doi: 10.7554/eLife.84392 (PMC10287166; doi:10.7554/eLife.84392)

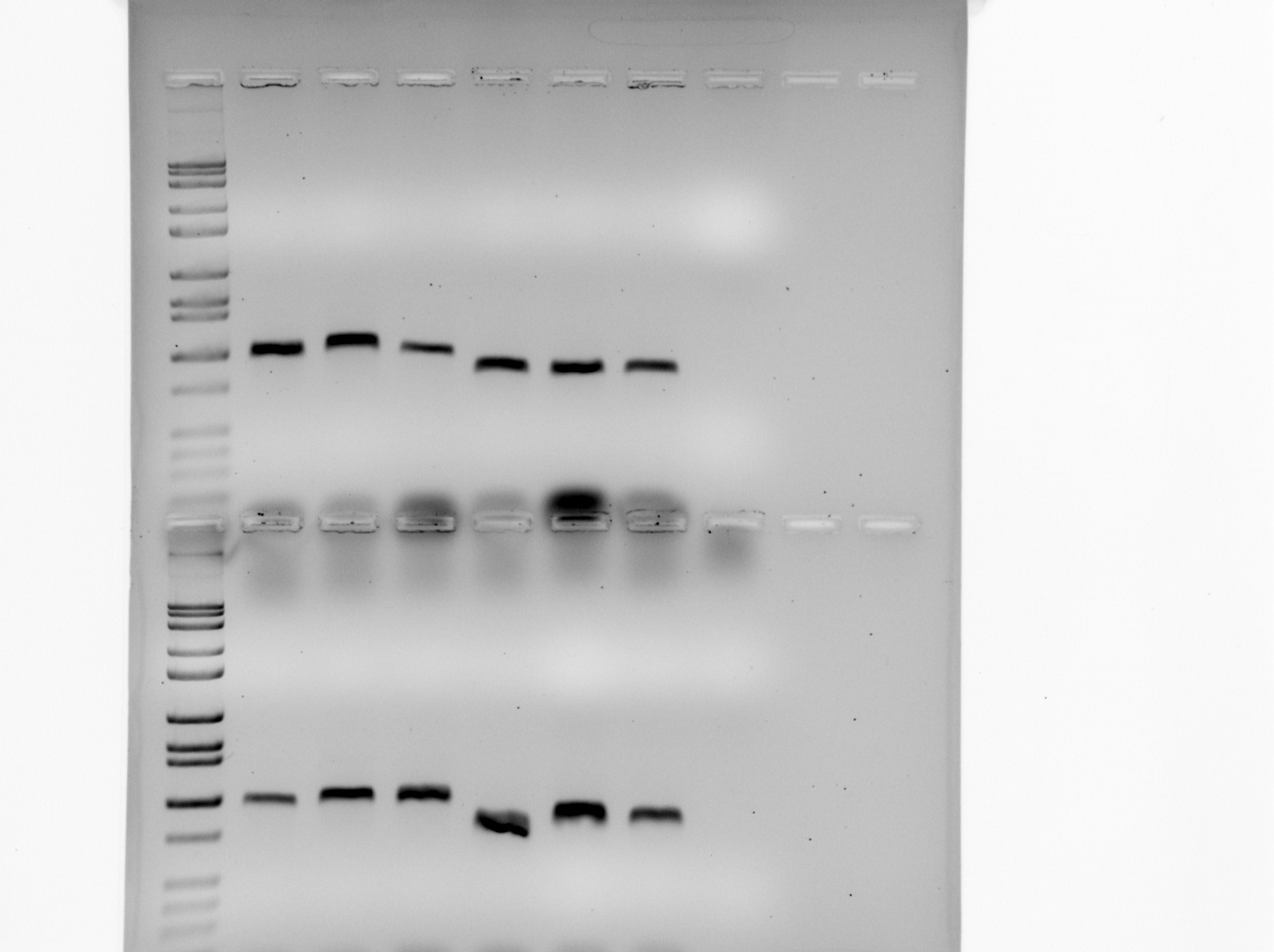

Supplement: Figure 3—figure supplement 1—source data 1. — Full unedited gel. [file elife-84392-fig3-figsupp1-data1.zip › Figure 3- figure supplement 2, source data 1.tiff]

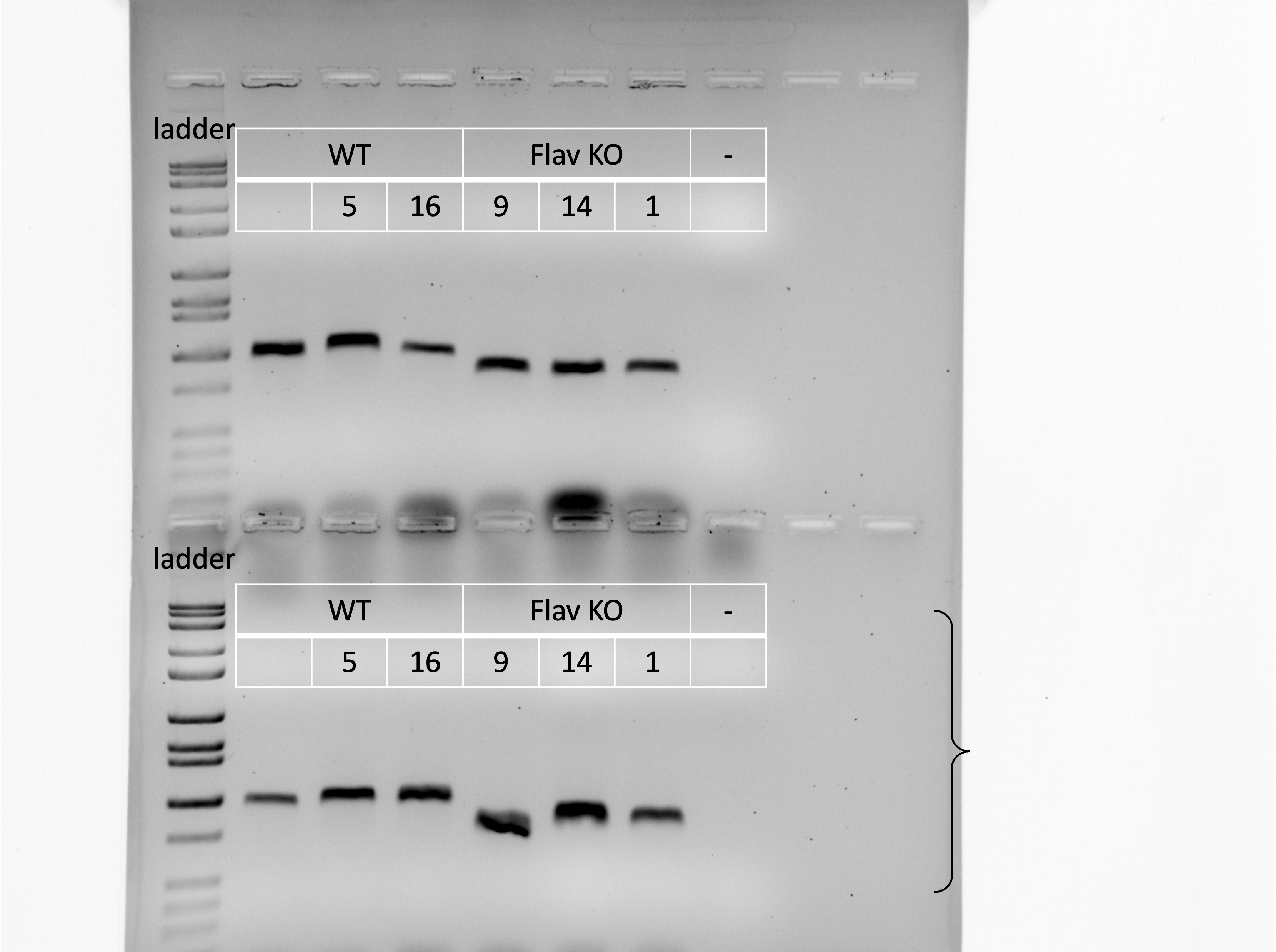

Supplement: Figure 3—figure supplement 1—source data 2. — Full gel with the bands clearly labeled. [file elife-84392-fig3-figsupp1-data2.zip › Figure 3- figure supplement 2, source data 2.png]
